# An Approach to Measuring Colistin Plasma Levels Regarding the Treatment of Multidrug-Resistant Bacterial Infection

**DOI:** 10.3390/antibiotics8030100

**Published:** 2019-07-24

**Authors:** Tatiana Pacheco, Rosa-Helena Bustos, Diana González, Vivian Garzón, Julio-Cesar García, Daniela Ramírez

**Affiliations:** 1 Evidence-Based Therapeutic Group, Clinical Pharmacology, Universidad de la Sabana, Chía 140013, Colombia; 2 Facultad de Medicina, Univesidad de La Sabana, Chía 140013, Colombia

**Keywords:** colistin, antimicrobial resistance, Gram-negative bacteria, therapeutic drug monitoring (TDM), multidrug-resistant (MDR), pandrug-resistant (PDR), extensive-drug resistant (XDR)

## Abstract

Antimicrobial resistance to antibiotic treatment has significantly increased during recent years, causing this to become a worldwide public health problem. More than 70% of pathogenic bacteria are resistant to at least one of the currently used antibiotics. Polymyxin E (colistin) has recently been used as a “last line” therapy when treating Gram-negative multi-resistant bacteria. However, little is known about these molecules’ pharmacological use as they have been discontinued because of their high toxicity. Recent research has been focused on determining colistimethate sodium’s pharmacokinetic parameters to find the optimal dose for maintaining a suitable benefit–risk balance. This review has thus been aimed at describing the use of colistin on patients infected by multi-drug resistant bacteria and the importance of measuring this drug’s plasma levels in such patients.

## 1. Introduction

Antimicrobial resistance (AMR) has represented a growing threat for several decades now regarding the effective treatment of an increasing range of infections caused by bacteria, parasites, viruses and fungi [1]. The development of AMR is a normal evolutionary process for all microorganisms, but it has undergone historic acceleration due to selective pressure exerted by the widespread/excessive use of antibacterial drugs. Several new antibacterial drugs had been developed by the 1970s to which most pathogens were completely susceptible. However, increasing AMR has been observed for the latest new families of antibiotics discovered around the 1980s, thereby leading to the rediscovery of old antibiotics such as colistin.

The World Health Organization (WHO) and the United States (US) Centres for Disease Control and Prevention (CDC) coincide in defining AMR as a microorganism’s natural or acquired ability to resist the effects of drugs having bactericide or bacteriostatic properties. Resistant bacteria survive exposure to such antibiotics and continue multiplying, causing more damage and their consequent spread to other hosts [2]. The aim of this article is to show the importance of therapeutic drug monitoring (TDM) regarding colistin.

## 2. Colistin Used in Managing MDR Infection

### 2.1. Colistin’s Chemical Structure

Polymyxins are natural products [3] and were isolated from soil bacterium *Paenibacillus polymyxa* subsp. *Colistinus* in 1947 [4] and identified as polymyxins produced by *Bacillus polymyxa var*. *Colistinus*. This drug has been available since 1959 for treating infection caused by Gram-negative bacteria.

Five main chemically different members have been recognised and designated polymyxins A, B, C, D and E [5]; two types of polymyxin are available for clinical use: polymyxin E (PME, i.e., colistin) and polymyxin B (PMB) [6]. PME has two particular forms, colistin sulphate (for oral and topical use) and negatively-charged *methanesulfonate (MSA) salt* of *colistin*, known as colistin methanesulfonate (CMS), or sodium colistimethate (SCM) in aerosol and injectable forms [5]. CMS is a poly-methanosulfonylated inactive prodrug of colistin and is not active microbiologically; it hydrolyses spontaneously to release PME [7].

**Figure 1:**
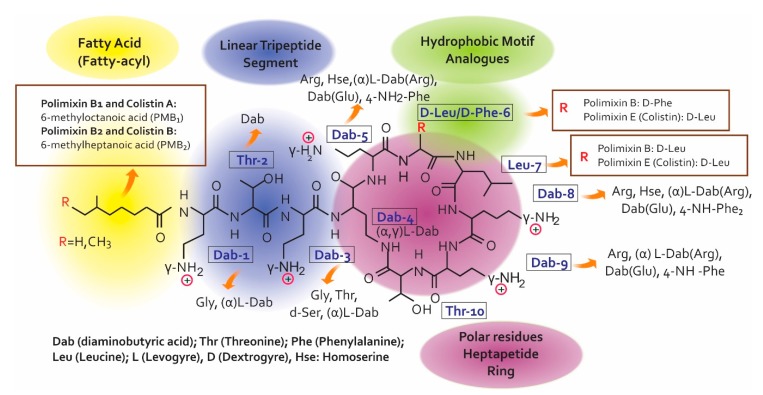
Chemical structure for colistin and polymyxin B [3,6,7,9,11].

Colistin’s chemical structure (Figure 1) defines its mechanism of action (MoA) and so understanding its structure-activity relationship (SAR) is an essential precursor for developing and discovering modern antibiotics [3]. Both PMB and PME are non-ribosomal secondary peptides from metabolites produced by soil bacterium *Bacillus polymyxa* which are highly bactericidal for Gram/negative bacteria. PMB is considered one of the most efficient permeabilising cell components [8]. The structural difference between both polymyxins occurs in position 6 which is occupied by _D_-Phe in PMB and _D_-Leu in colistin [9,10]. The polymyxin molecule has a hydrophobic structure consisting of an N-terminal fatty acid chain, five L-α, γ-diaminobutyric (Dab) acid residues, a linear tripeptíde segment, another hydrophobic structure in positions R6 and R7 on the cyclic heptapeptide ring and a heptapeptide main chain [3]. 

The decapeptide sequence contains the intramolecular cyclic heptapeptide in the Dab residue N-amino acid in position 4 and in the C-terminal threonine residue carboxyl group in position 10 [6]. It can be said from the description of polymyxin chemical structure that they are similar to that of the cationic antimicrobial peptides (CAMPs), representing the first line of defence against bacterial colonisation of eukaryotic cells [11]. Seven individual PMB have been identified to date. Six of the seven lipopeptides contain branched and unbranched N-terminal fatty acid groups, being structurally different regarding length by 7 to 9 carbons, called polymyxins B_1_–B_6_.

According to the SAR, polymyxins’ electrostatic and hydrophobic interactions with the lipid A component of Gram-negative bacteria’s outer membrane (OM) lipopolysaccharides (LPS) are essential in antimicrobial activity [12]. The polymyxin pharmacophore indicates that the positive charges of Dab 1, 3, 5, 8, 9 side chains represent the key points for such interaction; the pharmacophore is defined by the essential, steric, electronic and determination points of the function necessary for an optimal interaction with the pharmacological objective). The N-acyl fatty acid chain region and positions 6 and 7 on the heptapeptide cyclic ring form a fundamental part of the pharmacophore group. The positions of amino acids 2–5 and 8–10 show that they can become hydrogen donors. Another interesting aspect of polymyxin’s pharmacophore model is that it can be divided into polar (Dab and Thr residues) and hydrophobic domains (hydrophobic Na fatty acid chain and the D-Phe6-L-Leu7 motif). 

Polymyxins are produced by fermentation, mixtures being obtained which have more than 30 components [10]; there are differences between pharmacopoeias regarding some components’ limits. The European Pharmacopoeia (Ph. Eur.) has limits for some colistin and polymyxin components [13,14], whilst US Pharmacopeia (USP) has no limits [15,16]. Colistin’s inherent toxicity is due to the hydrophobicity of the N-terminal fatty acyl segment, thereby greatly influencing colistin-s antimicrobial activity. 

### 2.2. Mechanism of Action 

Knowledge of Gram/negative bacteria’s architecture is necessary when talking about the polymyxins’ MOA. The plasma membrane is the barrier protecting cells from agents which can be harmful for them, including numerous antimicrobials. Polymyxins’ antimicrobial action is exerted through direct interaction with the lipid A component of the LPS. Although several models based on biophysical studies have been proposed to date, the consensus reached so far has had to do with the interaction of lipid A as polymyxin binding target [6]. Colistin and PMB antibiotic activity is concentration-dependent and has little or no post-antibiotic effect. The MoA is based on disrupting the bacteria’s plasmatic membrane and an ability to bind to the LPS, thereby preventing the release of LPS components causing destruction outside/beyond the membrane [17].

Regarding the first mechanism, the polymyxin target for Gram-negative bacteria is outside the membrane [11]. The polymyxins act as detergent agents and disrupt membrane integrity, thereby causing structural changes due to its amphipathic nature, i.e., the hydrophilic portion provided by the peptides while the hydrophobic portion provides the acyl-fatty portion and the final part of the chain. Cell membrane damage increases permeability, causing all bacterial cells’ content to become lost, ending in cell lysis. The electrostatic interaction occurs between LPS anions and the antibiotic cations donated by Dab residues, causing release of divalent cations (Ca^2+^ and Mg^2+^) outside the membrane [11,18].

The second MoA is related to the antibiotic’s ability to bind to the LPS and endotoxin release. Considering that the LPS have an important function concerning bacteria, such as endotoxin release, the polymyxins neutralise LPS lipid A component [19]. Such neutralisation mainly arises from fatty acyls binding to the final part of the polymyxin structure’s tripeptide side chain. Polymyxins also neutralise LPS inhibitory effect on mononuclear cells’ transcriptional activity, thereby reducing proinflammatory cytokine release. This antibiotic also causes mast cell degranulation due to histamine release, thus facilitating cell apoptosis [17]. It has been observed that polymyxin causes sensitisation of Gram/negative bacteria’s cell membrane to other types of antibiotics such as fusidic acid, novobiocin, rifampicin and erythromycin [17].

### 2.3. Colistin Resistance Mechanisms

Excessive colistin use has caused bacterial resistance to this antibiotic (i.e., in bacteria which were where normally susceptible to it). Research on colistin has increased dramatically during the last decade [20]. Polymyxin resistance mechanisms have not all been elucidated and involve a number of regulatory systems; however, colistin resistance regarding Gram-negative bacteria has been attributed to different factors. The most documented colistin resistance mechanism concerning Gram-negative bacteria has been attributed to LPS modifications via diverse routes. The lipid A head groups reduce initial electrostatic interaction with *Escherichia coli*, *Salmonella enterica* serovar *Typhimurium*, *K. pneumoniae*, and *P. aeruginosa*. Modifying the phosphates of lipid A positively-charged groups, such 4-amino-4-deoxy-L–arabinose (L-Ara4N) and/or phosphoethanolamine (PE), decrease lipid A net negative charge, thereby producing resistance to polymyxins [6,9]. This effect is produced by other types of Gram-negative bacterial species (i.e., *S. enterica*, *E. coli*) and the regulatory systems of two-component PhoP-PhoQ and PmrA-PmrB (response regulator/sensor kinase), involving the *pmrAB*, *pmrD*, *phoPQ*, *parRS* and *mcr* genes/determinants (*Klebsiella pneumoniae* CG43). These systems regulate cationic AMR due to low environmental Mg^2+^ and Ca^2+^. 

Other two-component systems, such as ParR/ParS, ColR/ColS, and CprR/CprS [11,21], and alteration in the *mgrB* gene in *K. pneumonia* [22,23] (encoding a negative PhoP/Q regulator), cause structural modifications of the lipid A subunit, thereby affecting LPS charge and decreasing the electrostatic interaction between colistin’s negative and positive charges. Furthermore, the PmrA-PmrB system activates the expression of genes regulating lipid A modification enzymes. Another type of lipid A modification is associated with the loss of LPS, thus avoiding polymyxin fixation. Similarly, a loss of polymyxin target is caused by alteration in the *lpxA*, *lpxC*, and *lpxD* [24] genes, causing the inactivation of lipid A biosynthesis. It has been observed recently that a mutation in *CrrB (crrC)* is associated with *Klebsiella pneumonia* regarding colistin resistance [25]. Other genes have been identified as conferring resistance to polymyxin, such as *mcr-1* to *mcr-7 (E. Coli and K. pneumonia)* [25,26,27].

Another polymyxin repulsion mechanism results from adding D-alanine (D-Ala) to teichoic acids, thereby increasing net positive charge due to the *dlt-ABCD* genes. Adding D-Ala to an OM has been described in other bacteria, regulating *graXSR*, *dra*/*dlt*, *liaSR* and *CiaR* operons *(Staphylococcus aureus*, *Bordetella pertussis*, *Streptococcus gordonii*, *Listeria monocytogenes*, Group B *Streptococcus)* [26,27]. Additional mechanisms have been described inducing modifications on cell surface regarding electrostatic repulsion of colistin, such as lipid A deacylation and hydroxylation by genes encoding *Pag*, *PagL*, *LpxM* and *LpxO* enzymes and decreasing membrane fluidity/permeability [25]. Additional lipid A mechanisms include phosphorylation, dephosphorylation, glycylation and glucosylation [28], putative hopanoid and staphyloxanthin biosynthesis by *Bmul_2133/Bmul_2134 (Burkholderia multivorans)* and genes involved in staphyloxanthin biosynthesis.

Associated membrane remodelling resistance mechanisms include loss of polymyxin target and capsule polysaccharide (CPS) overproduction, thereby hiding polymyxin binding sites in *Neisseria meningitidis*, *Klebsiella pneumoniae and Salmonella enterica.* Altered membrane composition (*virB*, *suhB Bc*, *bvrRS*, *epsC-N*, *cgh*, *vacJ*, *waaL*, *rfbA*, *ompW*, *micF*, *pilMNOPQ operon*, *parRS*, *rsmA*, *bveA*, *ydeI (omdA)*, *ompD (nmpC)*, *ygiW (visP)*, *ompF*, *rcs* genes), altered membrane integrity (*cas9*, *tracrRNA*, *scaRNA*, *Lol*, *TolQRA* genes), lipooligosaccharide (LOS) and LPS modification (*spgM*, *pgm*, *hldA*, *hldD*, *oprH*, *cj1136*, *waaF*, *lgtF*, *galT*, *cstII*, *galU* genes) and loss of LPS and, consequently, loss of polymyxin target (*lpxACD*, *lptD*) are other membrane remodelling mechanisms [29].

Specific modifications to OM porins and overexpression of efflux pump systems have also been described. Various environmental factors such as oxidative stress, high temperature or salicylate affect porin expression through *micF* regulation [30,31]. Mutations in outer membrane porins (OmpU, OmpA and PorB) are associated with resistance to polymyxin B ([32]. Several types of multidrug efflux pumps (MtrC–MtrD–MtrE, RosAB, AcrAB–TolC (*Escherichia coli*), NorM, KpnEF and VexAB) play an important role in tolerance toward polymyxin B *(Neisseria gonorrhoeae)*. The marRAB operon acts through interactions with Rob and up-regulation of the AcrAB–TolC efflux pumps. Other efflux pumps have been described in polymyxin resistance, such as AdeABC, HlyD Mex pumps [33]. 

The collection of all antibiotic resistance genes (resistome) has been defined since 2006 as a framework for understanding the evolution of and emergency regarding resistance [34]. Recent colistin resistance studies have highlighted the gene (*dedA*) encoding putative integral membrane protein (DedA) as playing an important role in membrane homeostasis *(Escherichia coli)* [35]. DedA proteins are essential for bacterial viability; thus inhibiting DedA function may provide the basis for new antibiotic development [36,37]. Table 1 summarizes the main mechanisms of resistance of bacteria and their associated genes.

## 3. Clinical Management of Colistin

### 3.1. Pharmacokinetics (PK)

**Figure 2:**
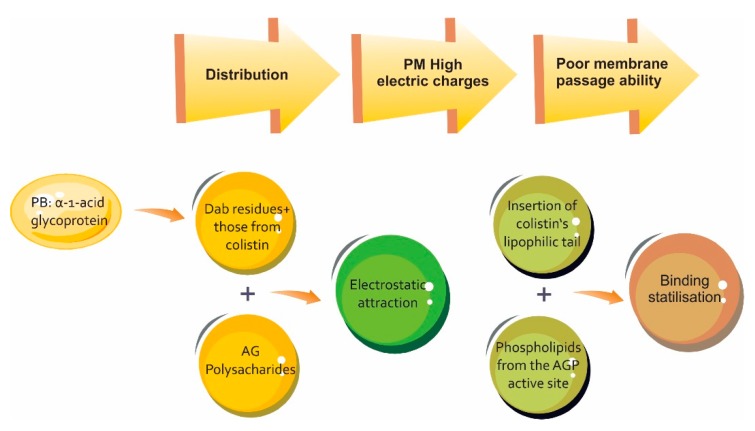
Schematic representation of binding properties of colistin for human α-1-acid glycoprotein (AGP) and the impact in the distribution process. PB: prote in binding [55].

One of the most important historical challenges in relation to colistin has been acquiring knowledge concerning its PK. Despite the discovery of this molecule more than six decades ago, and its resurgence due to the absence of a new therapeutic arsenal for MDR infections, the pharmacokinetic panorama is still not entirely clear. In this regard, about 70 articles are available in the pertinent literature, most of them dealing with (and coinciding on) this antibiotic’s pharmacokinetic variability which, associated with being a prodrug having a chemically complex structure and narrow therapeutic window, has thereby hampered in vivo and in vitro studies aimed at fully elucidating its behaviour. Figure 2 and Table 2; Table 3 summarise colistin distribution and protein binding.

Colistin elimination routes remain mostly unknown. Considering its peptide structure, colistin must be eliminated by hydrolysis, but the enzymes involved and their location are still unknown [53]. The blood, the liver and the kidneys are important sites for colistin elimination because they contain large amounts of proteases and peptidases; however, due to these enzymes’ ubiquitous availability throughout the body, colistin’s proteolytic degradation should not just be limited to the classical elimination organs. It is worth noting that colistin’s cyclical structure helps protect it from proteolytic endopeptidases and the acyl hydrophobic chain helps protect against exopeptidases, thereby explaining why colistin’s half-life is longer than that of many peptides [53]. This drug’s pharmacokinetic behaviour is specific for each type of population. The pertinent literature available in each case is summarised below.

#### 3.1.1. Healthy Volunteers

Only two studies have been published to date regarding this population. Couet et al. (2011) characterised CMS and colistin’s PK after intravenous (IV) administration of CMS in healthy young volunteers at the University Hospital of Poitiers’ Clinical Research Centre (France) [54]. The study involved twelve healthy young male volunteers aged 29.5 ± 5.5 years-old on average, having 72.7 ± 9.1 kg average body weight. The exclusion criteria consisted of heart, lung, liver, kidney, haematological, neurological and psychiatric diseases and severe obesity (defined as > 30 kg/m^2^ BMI (body mass index)). A single dose of 1 MIU (80 mg) CMS infused in 1 hour was used; multiple blood samples were taken from 0.5 h to 18 h after the start of infusion and urine samples between 0 and 12, 24 h after starting the dose [54]. The concentration profiles related to time elapsed in this study were parallel for CMS and colistin (Figure 2); however, it should be noted that colistin’s average lifespan was longer than that for CMS which would mean that colistin elimination was not limited by the speed of its formation [54]. Table 4 summarises this study’s findings.

Zhao et al., have also described colistin PK in healthy subjects in China; 24 volunteers were enrolled in their study which revealed that steady-state was rapidly achieved for colistin in healthy Chinese subjects using a 2.5 mg colistin base activity (CBA)/kg dose every 12 h. CMS half-life was much shorter than that for colistin. No significant CMS or colistin accumulation in plasma was observed within 1 week. This study characterised CMS and colistin urinary PK after 7-day treatment in humans; the very high concentration of colistin in urine strongly supported the use of IV CMS for serious urinary tract infections [65].

#### 3.1.2. Critically-ill Patients 

This molecule’s PK behaviour is more variable in this population. Table 5 summarises some general observations made in the pertinent literature. 

Significant discrepancies have arisen between available studies regarding this special population. Gregoire et al., observed typical C_max_ values after the first dose of 2 MIU (2 mg/L) CMS [67], whereas Plachouras et al., deduced 0.6 mg/L C_max_ for colistin after a first dose of 3 MIU CMS [68]. As can be observed, C_max_ values have been reached in around 3 h in a study by Gregoire et al. [67] unlike that of Plachouras et al., in around 8 h [68]. There have been fewer discrepancies between studies regarding steady-state; average steady-state colistin (Css,avg) was calculated as 1.5 to 3.5 mg/L for a patient having 82 mL/min creatinine clearance being treated with 3 MIU CMS every 8 h, depending on the study [68]. The aforementioned study was the first to highlight the difficulties in achieving an average 2 mg/L Css for patients having 80 mL/min creatinine clearance. This study thereby showed the probable relevance of the loading dose in reaching steady state in less time (i.e., a function of the drug’s elimination half-life - t_1/2_) and such theory has been corroborated in two further studies [58,69]. Average colistin C_max_ values were 1.3 mg/L (0.3–2.6 mg/L range) 8 h after dosing and t_1/2_ was 18.5 after administering 6 MIU CMS to ten critically-ill patients in the first of them [58]. After administering a 9 MIU loading dose to 19 critically-ill patients in the second study, colistin C_max_ values were also highly variable (mean 2.65 mg/L and 0.9–5.1 mg/L range) with 11.2 h t_1/2_ [69]. Menna et al., also concluded that a dose-intensified CMS regimen for critically ill patients suffering acute kidney injury (AKI) and continuous renal replacement therapy (CRRT) results in high and long-lasting colistin plasma levels [70]. These PK parameters have enabled dosage algorithms to be constructed; one of the most recognised in the literature is that which has been that proposed by Garonzik et al. [66].

#### 3.1.3. Patients having Extremely Impaired Renal Function

CMS is excreted sparingly in the urine and the dose fraction available for conversion to colistin is therefore higher [33]. Consequently, colistin exposure is generally three times greater in critically-ill patients requiring haemodialysis on days without a haemodialysis session than in patients having preserved renal function and being treated with the same dose of the drug [33]. Considering their molecular weights, CMS and colistin fractions not bound in plasma can thus pass freely through dialysis membranes [33]. Furthermore, colistin could also be adsorbed by dialysis membranes, especially those used for continuous renal replacement therapy, which could contribute towards the colistin clearance mechanism [31]. Table 6 summarises colistin and CMS clearance regarding dialysis mode. 

#### 3.1.4. Cystic Fibrosis Patients

Cystic fibrosis patients have been amongst those most studied to date due to this drug’s extended use in this population. Table 7 summarises some of the most important findings. 

#### 3.1.5. Burn Patients

Lee et al., have reported a 6.6 h colistin half-life following IV administration of 5 MIU CMS every 12 h, such clearance being comparable to that of critically-ill patients and healthy volunteers. This would suggest that it was not affected by this patient population’s hypermetabolism [74], as colistin Vd was slightly higher than that reported for healthy volunteers [74].

The currently available literature states that colistin PK vary widely which, associated with colistin being a drug having a narrow therapeutic index (NTI), makes the use of strategies such as monitoring plasma levels relevant after IV administration to ensure its proper use. The data regarding polymyxin safety is controversial. This antibiotic family has been historically associated with numerous adverse events (AE), including mortality, nephrotoxicity, neurotoxicity and hypersensitivity reactions [75].

One of the first reports regarding this drug’s safety was published in 1962, describing potentially serious reactions to colistin in 3 adults suffering renal failure and in a child who received ten times the recommended dose [76]. The first major study was published in June 1970 which evaluated adverse reactions in 317 courses of CMS therapy; prior to this, it was considered that CMS toxicity was low. Some researchers declared in the 1960s that using this drug did not induce nephrotoxicity; however, this investigation’s results alerted the global medical community, adverse reactions being seen in 1 out of every 3 patients [77].

From the above and other findings around the same time, compounds from this class of antibiotic were gradually withdrawn from clinical practice and replaced by newer antibiotics having the same or broader antibacterial spectra and whose toxicity reports were lower [78].

The association between polymyxins and mortality represents one of the most controversial issues regarding this topic; two major meta-analyses in this regard have assessed mortality in two pneumonia patient populations associated with mechanical ventilation. One sampled 1167 and the other 796 patients; no differences were found in both regarding all causes of death when comparing patients treated with colistin to those treated with other antibiotics. However, in neither study was it clear whether polymyxins can contribute to total mortality through their nephrotoxicity [79,80]. Later, in 2010, Falagas et al., and Elias et al., found a protective polymyxin dose effect, despite the development of acute kidney injury [81,82]. This has been corroborated by other studies showing greater area under curve (AUC)/minimum inhibitory concentration (MIC) exposure at higher polymyxin doses, ultimately predicting better activity for these drugs [83,84]. However, studies are required having a better methodological design involving significant variables affecting mortality after 30 days’ treatment [75].

Renal toxicity is the most commonly occurring AE related to polymyxin use, ranging from proteinuria to acute renal injury requiring interruption of therapy and even the initiation of renal replacement therapy; overall incidence has high (20% to 76%) variability based on updated data [75]. Recent studies have shown lower incidence regarding this AE than that thought in previous decades. The Greek research group led by Falagas has shown that deterioration in patients having normal basal creatinine was not significant during prolonged colistin administration, thereby contrasting with other available studies; they did not even find these doses’ influence on the renal function of patients having prior dysfunction [85]. Hartzell et al., have shown that 21% of cases have required cessation of therapy, but that no patient had to undergo renal replacement therapy [86].

Several theories have been advanced regarding polymyxins’ renal toxicity. It has been assumed that this may have been partly due to their content in D-amino acid isomeric form and the acid fatty component. Experimental studies have shown increased trans-epithelial conductance through the bladder’s epithelium [87]; CMS can then induce greater membrane permeability, resulting in cell lysis [87,88]. Between 49% and 78% of cases of nephrotoxicity occur within the first 5 to 7 days of therapy [89]. The risk factors related to this AE are numerous; however, most data comes from retrospective studies and must be interpreted carefully. One of the main controversies has thus concerned the dose; some studies have confirmed a relationship between nephrotoxicity and high total cumulative doses or longer-lasting therapy whilst others have failed to confirm such observations [75]. Other risk factors identified so far have been related to patients’ age [90] and the concomitant use of other nephrotoxic drugs [91]. Kady Phe (2014) compared and validated the performance of several models for predicting the risk of colistin-associated nephrotoxicity, identifying age, therapy duration and daily dose according to ideal weight as independent risk factors. Interestingly, cystic fibrosis was found to be a protective factor, no association being found with the concomitant use of other nephrotoxic agents [92]. Mathematical prediction models will undoubtedly prove useful for correctly identifying high-risk patients so that strategies aimed at minimising such AE can be selectively introduced; however, studies having better methodological design are required to unify data regarding polymyxin-associated nephrotoxicity.

A rather less-reported relevant AE in the literature has been neurological toxicity; its overall incidence is less than 7% and even recent multiple studies have not reported any cases [75]. Paraesthesia has been the most frequently reported neurotoxic effect amongst the wide range of such effects (7% to 23%), being even higher in cystic fibrosis patients [78,93]. Reports of other neurotoxic events are scarce, only three cases of respiratory/ventilatory failure having been reported after 1970 [94]. This could have been related to the difficulty regarding an objective interpretation of neurological symptoms which would have influenced clinical nephrotoxicity and neurotoxicity reports in not identifying or reporting polymyxin-associated neurotoxicity [75]. Direct interaction between polymyxins and neurons is considered the cause of neurotoxicity; this could lead to inhibiting acetylcholine action regarding neuromuscular binding, increase depolarisation and induce histamine release. However, the precise mechanism has not yet been completely elucidated [75]. Renal dysfunction accompanied by concomitant neurological diseases, such as *myasthenia gravis*, have been identified as risk factors [95].

The available literature regarding polymyxins’ other toxic effects is scarce; it is not clear however, whether their low incidence is real or whether one is dealing with these events’ slight clinical relevance or bias in the available retrospective studies [75].

## 4. Colistin Plasma Level Measurements

This drug’s dose and levels in blood must be controlled due to the increasing use of last-line antibiotics, especially polymyxins and mainly colistin [96]. Colistin dosing regimens derived from cases of acute Gram-negative bacterial infection have been based on clinical experience for decades now, thereby leading to doses not being defined by the antibiotic’s pharmacodynamic (PD) and PK properties [97], due to the lack of specific and reliable methods for taking measurements [98]. Robust assays have thus become necessary to enable the antibiotic to be quantified for determining its dose and mitigate its AEs regarding patients’ health by introducing/using techniques enabling this drug to be measured using different matrices, like milk, saliva, blood, plasma and tissue [99].

### 4.1. Analysis Methods

Several techniques have been described so far which have focused on quantifying CMS in plasma, including microbiological bioassays mainly based on the drug inhibiting microorganisms using pathogenic bacteria from the *Bordetella bronchiseptica* and *Escherichia coli* genera as indicator [100,101]. High-performance liquid chromatography (HPLC) is currently one of the most used analysis techniques; it has a high degree of accuracy and precision based on separating a sample’s components, involving different types of chemical interaction [100]. Mass spectrometry (MS) is a highly selective and sensitive technique, thereby enabling colistin quantification in different matrices, especially human plasma [102]. This drug’s molecule has little ultraviolet (UV) absorption and no fluorescence, which is why techniques such as UV spectroscopy are not frequently used for its quantification [103]. Table 8 shows the analytical method used for determining colistin in plasma. 

### 4.2. Difficulty Regarding Measurement

Difficulties have been encountered when measuring colistin effectiveness [104]. One of the main problems has arisen regarding colistin’s chemical characteristics as it is adsorbed by many materials used in the laboratory, such as plastic. Colistin’s structure is affected by an experiment’s physical-chemical conditions, mainly sample temperature, pH, incubation time and matrix, thereby leading to CMS degradation (hydrolysis) to colistin [97,104]. Such experimentation difficulties have led to false positives when quantifying the drug.

Some analysis- and quantification-related disadvantages must be highlighted regarding the techniques used to date for measuring colistin levels, thereby hampering reliable results being obtained. Microbiological bioassays represent one of the most inaccurate and least sensitive techniques because the incubation conditions interfere with molecule stability [100]. This technique involves using culture media which are difficult to obtain on the market; furthermore, the variables regarding microorganism growth can affect assay results [101].

HPLC has been widely used since it is a more sensitive and precise technique than a microbiological bioassay. However, its disadvantage lies in being a test which requires expensive equipment, high reagent consumption, sample pre-treatment, solid phase extraction and derivatisation which requires specialised personnel [108,109]. Difficulties have arisen regarding analysing and quantifying the mixture of CMS compounds in its commercial presentation, thereby hindering their separation [100].

MS is one of the most robust techniques for measuring colistin levels; however, it requires a specialised laboratory and trained personnel for analysing and quantifying samples, bearing in mind that the sample is destroyed during the process [68]. The molecules are derivatised when using other techniques, such as UV spectroscopy; reagents such as 9-fluorenylmethyl (FMOC-Cl) and ortho-phthalaldehyde (OPA) are used in their analysis and quantification [97].

### 4.3. The Importance of Measurement

Limited data regarding colistin PK and PD properties has led to confusion when determining patients’ doses, added to the uncontrolled increase of colistin-resistant bacteria and their neurotoxic and nephrotoxic effects [110]. The forgoing highlights the importance of monitoring colistin plasma levels to adjust the dose and dose interval, taking a particular patient’s clinical picture into account [111]. PK properties may become altered in critical patients because they are frequently prone to large oscillations regarding distribution volume, renal clearance fluctuations and protein binding variability. Likewise, these drugs’ antimicrobial activity is attenuated by the high bacterial load, as in pneumonia [112].

Different techniques, involving a high degree of sensitivity and precision, have thus been used for enabling colistin plasma concentrations to be quantified [113]. However, most are expensive and require specialised personnel and laboratories whilst matrix components and physico-chemical conditions can interfere with assay accuracy [114]. Nevertheless, this drug must be quantified for determining an appropriate dose mitigating resistance to it and its toxicity [97].

## 5. Conclusions

Although, there is still not enough evidence regarding adjusting an antibiotic dose for patients suffering multi-resistant bacterial infection, therapeutic monitoring of colistin could constitute good clinical practice to help administer a dose to patients according to infection levels and drug response. Clinical use of intravenous colistin is limited by its large interpatient PK variability; some dosing algorithms have been constructed in an attempt to define a desirable concentration. However, plasma concentrations overlapping for an antibacterial effect and those causing nephrotoxicity and neurotoxicity limit its feasible implementation.

## Figures and Tables

**Table 1:** Strategies employed by bacteria for achieving resistance to colistin.

Genes Involved	Resistance Mechanism	Bacteria	Ref
**LPS modifications**			
*arn*BCADTEF operon and *pmrE*	Modification of the lipid A with aminoarabinose	*Salmonella enterica*, *Klebsiella* *pneumoniae*, *Escherichia coli*, *Proteus mirabilis*, *Proteeae bacteria*, *Serratia* *marcescens* and *P.* *aeruginosa*	[38,39,40,41,42,43,44,45]
*crrB (crrC)*	The regulatory systems of two-component PhoP-PhoQ and PmrA-PmrB (response regulator/sensor kinase)	*K. pneumoniae* CG43	[25,46]
*pmrAB*, *pmrD*, *phoPQ*, *parRS*, *mcr*	L-Ara4N and PEtn modification of lipid A	*E. coli*, *Salmonella enterica*, *P. aeruginosa*	[25,40,47]
*ParR/ParS*, *ColR/ColS* and *CprR/CprS*	LPS modification with aminoarabinose	*P. aeruginosa*	[11,21]
*mgrB*	Structural modifications of the lipid A subunit	*K. pneumonia*	[22,23]
*lpxACD*, *lptD*	Loss of LPS	*Acinetobacter baumannii*	[24,48]
*pmrC*	Modification of the lipid A phosphoethanolamine	*S. enterica*, *K. pneumoniae*, *E. coli* and *Acinetobacter baumannii*	[38,49]
*mcr-1 to mcr-8*	Inactivation of lipid A biosynthesis	*E. Coli* and *K. pneumonia*	[11,50]
*Pag*, *PagL*, *LpxM* and *LpxO*	Modifications on cell surface regarding electrostatic repulsion of colistin Decreasing membrane fluidity/permeability	*K. pneumoniae*, *E. coli*, *S. enterica* and *Legionella pneumophila*	[25,51]
*Bmul_2133/Bmul_2134*	phosphorylation, dephosphorylation, glycylation and glucosylation of lipid A	*Burkholderia multivorans*	[28,52]
**Repulsion mechanism**			
*dlt-ABCD*, *graXSR*, *dra*/*dlt*, *liaSR* and *CiaR* operons	Adding D-alanine (D-Ala) to teichoic acids, thereby increasing net positive charge	*Staphylococcus aureus*, *Bordetella pertussis*, *Streptococcus gordonii*, *Listeria monocytogenes* and Group B *Streptococcus*	[53,54]
**Membrane remodelling**			
*siaD*, *cps operon*, *ompA*, *kpnEF*, *phoPQ* and *rcs*	Loss of polymyxin target and capsule polysaccharide (CPS) overproduction	*Neisseria meningitidis*, *K. pneumoniae* and *S. enterica*	[40]
*virB*, *suhB Bc*, *bvrRS*, *epsC-N*, *cgh*, *vacJ*, *waaL*, *rfbA*, *ompW*, *micF*, *pilMNOPQ operon*, *parRS*, *rsmA*, *bveA*, *ydeI (omdA)*, *ompD (nmpC)*, *ygiW (visP)*, *ompF*, *rcs*	Altered membrane composition	*Brucella ovis*, *S. enterica*, *Brucella melitensis*, *Burkholderia cenocepacia*, *Vibrio cholerae*, *Brucella abortus*, *K. pneumoniae*, *P. aeruginosa*, *N. meningitidis* and *Brucella melitensis*	[29,40]
*cas9*, *tracrRNA*, *scaRNA*, *Lol*, *TolQRA*	Altered membrane integrity	*Stenotrophomonas maltophilia*, *Vibrio fischeri*, *B. cenocepacia*, *E. coli*, *P. aeruginosa*, *Salmonella Typhimurium*, *Campylobacter jejuni* and *Haemophilus influenzae*	
*spgM*, *pgm*, *hldA*, *hldD*, *oprH*, *cj1136*, *waaF*, *lgtF*, *galT*, *cstII*, *galU*	Lipooligosaccharide (LOS) and LPS modification		
**Modifications to OM porins and overexpression of efflux pump systems**			
*OmpU*, *OmpA and PorB*	Mutations in outer membrane porins	*N. meningitidis* and *V. cholerae*	[32]
*MtrC –MtrD –MtrE*, *RosAB*, *AcrAB–TolC*	An important role in tolerance toward polymyxin B	*E. coli*	[33]
*NorM*, *KpnEF and VexAB*		*Neisseria gonorrhoeae*	
*dedA*	playing an important role in membrane homeostasis	*E. coli*	[34,35,36,37]

**Table 2:** Colistin binding to proteins reported to date.

Colistin Binding (CB)	Population	Reference
55%	Dogs, calves	[56]
91%	Mice	[57]
59%–74%	Critically ill patients	[58]

**Table 3:** Colistin distribution in tissues.

Tissue	Characteristics	Ref
The lungs	Imberti et al., could not measure colistin in bronchoalveolar lavage (BAL) after repeated IV doses of 2 million international units (MIU) CMS every 8 h to critically-ill patients. Boisson et al., reported 0.1 and 29 mg/L colistin concentrations in steady-state epithelial lining fluid (ELF). Yapa et al., reported lower than 1 mg/L colistin concentrations in sputum after a single IV dose of 5 MIU CMS. No active transport has yet been reported for colistin’s passage across the pulmonary barrier.	[59] [60] [61]
The central nervous system (CNS)	Passage across the blood-brain barrier BBB becomes limited after repeated IV doses (<5%) in critically-ill patients. Inflamed meningeal membranes increased to 11% concentration in cerebrospinal fluid (CSF), even greater when administered by intrathecal route. CSF concentrations vary between 0.6 and 1.5 mg/L when patients are treated with IV 3 MIU CMS every 8 h plus intra-ventricular 0.125 MIU CMS every 24 h.	[62,63,64]
Peritoneal liquid	A case report has been published regarding a patient suffering severe peritonitis following multiple administrations of 2 MIU CMS every 8 h. Colistin became slowly distributed in the peritoneal fluid but colistin concentrations in peritoneal fluid were similar to that of steady-state plasma.	[63]

**Table 4:** Colistin’s pharmacokinetic parameters regarding healthy volunteers [54].

Parameter	Colistimethate	Colistin
Cmax (μg/mL)	4.8	0.83
Tmax (h)	-	2.0
**Distribution**		
Vd	Vc: 8.92 L Vss: 14 L	12.4 mL/min
**Elimination**		
CL (mL/min) ErCL RCL	148 48 103	48.7 46.6 1.9
t1/2 (h)	0.49	3.0

CL: clearance; ErCL: extrarenal space clearance; RCL: renal clearance; Cmax, maximum/peak concentration; t1/2: half-life; Tmax: maximum/peak concentration time; Css,avg: average steady-state plasma colistin concentration; Vd: volume of distribution; Vc: volume of central compartment distribution; Vss: steady-state volume of distribution; PB: protein binding.

**Table 5:** General PK aspects regarding maintenance dose in critically-ill patients.

With Maintenance Dose	References
-C_max_ has been observed at the end of the infusion. -Concentrations have become reduced mono-or bi-exponentially -t1/2 = 1.9–4.5 h -Typical CMS renal clearances for patients having 120, 50 and 25 mL/min creatinine clearance values has been around 100, 50 and 25 mL/min, respectively. -CMS fraction converted into colistin has increased by 33%, 50% and 67% for each value, resulting in higher colistin concentrations for patients suffering impaired renal function.	[66] [67]

**Table 6:** Average colistin and CMS clearance by dialysis mode.

	CMS Clearance	Colistin Clearance	Reference
Intermittent haemodialysis	71 to 95 mL/min	57 to 134 mL/min	[71,72]
Continuous venovenous haemofiltration (CVVH)	64 mL/min	34 mL/min	[66]
Continuous venovenous haemodiafiltration (CVVHDF)	--	50%	[29]

**Table 7:** Pharmacokinetic parameters regarding cystic fibrosis patients.

Parameter	Characteristics	Reference
CL|	100 mL/min	[61]
Vd	18 L	[73]
T1/2	2.5 h	[73]
Exposure	>39% than in healthy volunteers	[61]

**Table 8:** Techniques used for quantifying colistin in plasma.

Technique	Methodology	Results	Ref
Microbiological bioassays	Quantifying colistin in human plasma using *E. coli* as indicator organism	Bioassays have mainly been used regarding clinical samples—evaluating urine and serum samples—less sensitive and specific tests	[100]
Fourier-transform infrared spectroscopy (FTIR)	Direct quantification of colistin methanesulfonate by attenuated total reflectance (ATR) FTIR	FTIR has enabled colistin to be detected in human plasma but must be complemented with other techniques, such as HPLC	[32]
High-resolution liquid chromatography (HPLC)	HPLC validation using fluorescence detection assay for quantifying colistin in plasma samples from hospitalised patients	A C18 column has been used with a mobile phase consisting of acetonitrile and water having a shorter retention time. Furthermore, this method has successfully quantified total colistin in plasma from patients treated with CMS	[30]
	Quantifying colistin in plasma from *Pseudomonas aeruginosa*-infected mice	Accuracy and reproducibility have ranged from 10.1% to 11.2% with rat and urine plasma, respectively. Several antibacterial agents which have often been administered together have not interfered with the assay	[104]
HPLC with evaporative light scattering detector (ELSD)	Quantifying colistin in plasma by HPLC with an ELSD	The method has proved to be specific, accurate, precise and linear	[105]
Diode array HPLC detector	Quantifying colistin in animal plasma by HPLC with diode array detector	Scanning in the UV 200-380 nm range, 206 and 208 nm wavelengths have enabled colistin to be quantified	[106]
Liquid chromatography mass spectrometry (LC-MS)	Routine quantification of colistin A and B and their respective CMS A and CMS B prodrugs in human plasma and urine	Pre-validation studies have demonstrated CMS stability in biological samples and extracts, this being a key point regarding reliable quantification of colistin and CMS. The assay has proved precise/accurate and reproducible for quantifying colistin A and B and CMS A and B in plasma samples	[102]
Ultra-performance liquid chromatography-electrospray tandem mass spectrometry with electrospray ionisation (UPLC-ESI-MS/MS)	Quantifying colistin in human plasma by a combination of techniques UPLC-ESI- MS/MS	Validation results have shown that the method had suitable selectivity and sensitivity. The method has been successfully used with plasma samples from cystic fibrosis patients who have been treated with colistin. The PK profile has been calculated.	[107]
